# Transcriptome Screening of Hormone-Regulated Genes Related to Fruit Development in *Zizyphus jujuba* Mill. cv. Goutou Fruits at Different Ripening Stages

**DOI:** 10.3390/ijms26083476

**Published:** 2025-04-08

**Authors:** Shuting Luo, Yusen Zhang, Beibei Shi, Rui Wang, Ziyan Zhang, Jiawen Wu, Zhenqing Bai, Guoliang Chen

**Affiliations:** 1 College of Life Sciences, Yan’an University, Yan’an 716000, China; 2 Inner Mongolia Academy of Science and Technology, Hohhot 010018, China; 3 Shaanxi Key Laboratory of Research and Utilization of Resource Plants on the Loess Plateau, College of Life Sciences, Yan’an University, Yan’an 716000, China

**Keywords:** *Zizyphus jujuba* Mill. cv. Goutou, transcriptomic, plant hormones, ripening

## Abstract

*Zizyphus jujuba* Mill. cv. Goutou is an edible and medicinal fruit whose development significantly impacts the metabolism and accumulation of nutrients and is regulated by plant hormones. In this study, the metal element and triterpene acid content were investigated and transcriptomic analyses were conducted to evaluate changes in seven ripening stages (Stages I–VII) of *Z. jujuba* Mill. cv. Goutou. We first analyzed seven metal elements and found that the concentrations of Magnesium (Mg), Aluminum (Al), Calcium (Ca), Manganese (Mn), and Cuprum (Cu) were highest at Stage I; in comparison, the concentrations of Zinc (Zn) and Ferrum (Fe) were highest at Stage IV. Additionally, three triterpene acids were detected in the fruits, with the contents of betulinic acid and oleanolic acid being highest at Stage VII and that of ursolic acid being highest at Stage I. Subsequently, through Gene Ontology (GO) and Kyoto Encyclopedia of Genes and Genomes (KEGG) annotations, we identified 40, 18, 9, 6, and 11 differentially expressed genes involved in the auxin, abscisic acid (ABA), ethylene, gibberellic acid (GA), and jasmonic acid (JA) signaling pathways, respectively. Notably, genes associated with ABA, including *ZjABA3*, *ZjABA4*, *ZjABA6*, *ZjABA7*, *ZjABA10*, *ZjABA11*, *ZjABA15-ZjABA19*, *ZjABA22-ZjABA25*, and *ZjABA27-ZjABA33*, were downregulated from Stage I to Stage VII. Conversely, the expression of *ZjACO* in the ethylene signaling pathway was the highest at Stage VII. *ZjMYC2-1*, a JA signaling pathway gene, was significantly induced at Stage I compared to in the other stages. The genes *ZjGID-1* and *ZjTF-1*, related to GA, exhibited the highest expression levels at Stage VI.

## 1. Introduction

*Zizyphus jujuba* Mill. cv. Goutou, a member of the Rhamnaceae family, *Ziziphus* Mill, is predominantly located in Yan’an, Shaanxi Province, China. The contents of metabolites in jujube fruits are crucial indicators for assessing the quality of fresh jujubes and the quality of these fruits is related to ripening. Triterpenes and metal elements are important nutritional components in jujube fruits [1]. Metal elements play crucial roles in human health and are also involved in plant growth. *Z. jujuba* Mill. cv. Goutou fruits contain abundant metal elements, including Fe, Cu, Mg, K, Ca, and Zn [2]. Fe is recognized for its blood-enriching properties. Research indicates that Fe acts as a co-factor for redox enzymes that influence its activity, with these enzymes inducing changes in fruit color [3]. Zn is recognized for its role in strengthening the immune system and promoting the synthesis of plant auxins [4]. In one study, the spraying of Ca increased the hardness and elasticity of the fruit peel, reducing the occurrence of fruit cracking [5]. The triterpene acids in jujube fruits mainly consist of pentacyclic triterpenoids, with oleanolic acid, ursolic acid, and betulinic acid being the main active components [6]. Researchers have found that these three compounds possess hepatoprotective properties and increase leukocyte activity, boost immunity, and exhibit anti-cancer effects [7]. Additionally, triterpenes also regulate the growth, development, and reproduction of plants [8].

The ripening of jujube fruits is regulated by endogenous plant hormones [9], including ethylene, abscisic acid (ABA), gibberellic acid (GA), and jasmonic acid (JA) [10]. ABA binds to the receptor pyrabactin resistance 1-like (PYL) and induces a conformational change, with it forming a PYL-ABA complex with protein phosphatase 2C (PP2C). This complex prevents the dephosphorylation of Sucrose Non-Fermenting 1-Related Protein Kinase 2 (SnRK2) and activates downstream effector proteins through kinase-mediated phosphorylation [11]. Exogenous ABA promotes the synthesis of endogenous ABA and ethylene and impacts the dynamic balance between ABA, GA, and ethylene to promote the synthesis of anthocyanins, which influence the color of tomato fruits [12]. The color of *Z. jujuba* Mill. cv. Goutou fruits at different ripening stages exhibits significant differences [13], and endogenous ABA is positively correlated with the ripening of jujube fruits [14]. In one study, the spraying of ABA promoted the accumulation of Mn in tomato fruits [15]. In another study, low concentrations of ABA induced the expression of the key enzyme gene *BpSE*, which is involved in triterpene synthesis in birch [16].

Ethylene binds to the ethylene receptor (ETR) to act on the endoplasmic reticulum, which subsequently interacts with the protein kinase Copper Transport Protein 1 (CTR1) to inhibit the membrane protein Ethylene insensitive 2 (EIN2). EIN2 influences the downstream transcription factor Ethylene insensitive 3 (EIN3), which is also subjected to inhibition by EIN3-binding F-box protein 1 and 2 (EBF1/2). The expression of EIN3 directly regulates the Ethylene response factor 1/2 (ERF1/2) and influences the ripening of fruits [17]. The results of previous studies show that ethylene mediates the dehydration and softening processes during the storage of kiwifruit, with exogenous Monocyte Chemoattractant Protein-1 (1-MCP) delaying kiwifruit ripening by inhibiting ethylene receptors [18].

Through jasmonate-resistant 1 (JAR1), JA is conjugated to JA-Ile, which directly interacts with JASMONATE-ZIM DOMAIN protein (JAZ) and coronatine-insensitive 1 (COI1) to activate Myelocytomatosis protein (MYC) transcription factors, leading to the degradation of JAZ via the 26S proteasome pathway and influencing the ripening of fruits [19]. Following treatment with exogenous JA in peach fruits, the production of ethylene was found to increase, the decline in soluble sugar content decreased, and the speed of fruit browning was effectively slowed, and the peach’s resistance to cold stress was improved [20]. In another study, the application of methyl jasmonate (MeJA) induced the expression of *SlJIG* to regulate terpenoid biosynthesis [21]. Furthermore, treatment with exogenous MeJA slowed the senescence of jujube fruits, enabling the effective maintenance of fruit firmness [22]. GA is recognized by the GA receptor Gibberellin-insensitive dwarf (GID1), which binds to DELLA proteins, negative regulators in the GA signaling pathway. After binding to GID1, the inhibitory effect of DELLA (aspartic acid–glutamic acid–leucine–leucine–alanine) proteins was alleviated, allowing GA signaling to regulate various physiological processes in the plant [23]. GA is involved in the development of sand pears [24].

In this study, the metal element and triterpene acid contents of *Z. jujuba* Mill. cv. Goutou fruits at different ripening stages were investigated to evaluate their nutritional value. Furthermore, we conducted transcriptome sequencing of *Z. jujuba* Mill. cv. Goutou fruits at different ripening stages. Combined with Gene Ontology (GO) and Kyoto Encyclopedia of Genes and Genomes (KEGG) pathway annotations, differentially expressed genes were identified that are involved in the ABA, ethylene, JA, and GA signaling pathways. Real-time quantitative PCR (RT-qPCR) was used to validate the expression levels of the differentially expressed genes and comprehend their roles in the regulatory mechanisms of development in *Z. jujuba* Mill. cv. Goutou fruits. Hormonal regulation significantly influences fruit development, impacting maturation, flavor, storage potential, and coloration, ultimately affecting fruit growth and quality. Investigating key genes involved in fruit maturation-related hormone pathways allows for the optimization of fruit development and storage through the genetic manipulation of hormone levels or receptor expression. The results presented in this study provide a theoretical basis for improving the quality and breeding strategies of *Z*. *jujuba* Mill. cv. Goutou.

## 2. Results

### 2.1. Detection of the Metal Element, Oleanolic Acid, Betulinic Acid, and Ursolic Acid Content of the Z. jujuba Mill. cv. Goutou Fruits at Different Ripening Stages

**Figure 1:**
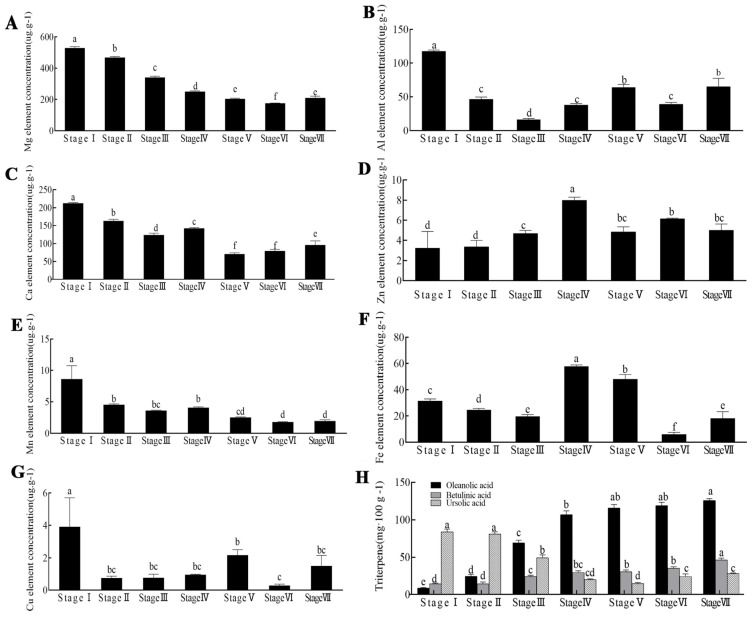
The metal element, oleanolic acid, betulinic acid, and ursolic acid content of *Z. jujuba* Mill. cv. Goutou fruits at seven ripening stages. (**A**) Mg element concentration (μg g^−1^); (**B**) Al element concentration (μg g^−1^); (**C**) Ca element concentration (μg g^−1^); (**D**) Zn element concentration (μg g^−1^); (**E**) Mn element concentration (μg g^−1^); (**F**) Fe element concentration (μg g^−1^); (**G**) Cu element concentration (μg g^−1^). The different small letters indicate a significant difference (*p* < 0.05); (**H**) the oleanolic acid, betulinic acid, and ursolic acid content of *Z. jujuba* Mill. cv. Goutou fruits at seven ripening stages. The error bars indicate the standard errors (*n* = 3) of the data.

*Z. jujuba* Mill. cv. Goutou contains a wide range of trace metal elements (Figure 1). During *Z*. *jujuba* Mill. cv. Goutou maturation, the Mg content gradually decreased, and the lowest concentration was detected at Stage VI compared to the other stages (Figure 1A). The Al content was highest at Stage I and showed a decreasing then increasing trend during the maturation of the fruit. No significant differences in Al content were detected between Stages II, IV, and VI, nor between Stages V and VII (Figure 1B). The Ca content was higher at Stages I–IV than at Stages V–VII (Figure 1C). The Zn content was highest at Stage IV (Figure 1D). The Mn content was highest at Stage I and showed a significant decrease during jujube ripening (Figure 1E). The Fe content was relatively higher at Stages IV and V, with significant differences observed among the other stages (Figure 1F). The Cu content was significantly higher at Stage I compared to all other stages (Figure 1G). Overall, the Mg, Ca, and Mn element content generally showed a decreasing trend.

The analysis of the three triterpenic acids at the seven ripening stages of *Z. jujuba* Mill. cv. Goutou is shown in Figure 1H. The oleanolic acid content ranged from 8.44 to 125.76 mg/100 g, with the highest level observed at Stage VII. The betulinic acid content ranged from 14.30 to 46.57 mg/100 g, with the highest level observed at Stage VII, with no significant differences between Stages I and II, Stages III and IV, and Stages V and VII. The ursolic acid content ranged from 14.60 to 83.80 mg/100 g, with the highest level observed at Stage I, and exhibited a decreasing then increasing trend. In summary, oleanolic and betulinic acid reached their highest concentrations at Stage VII, whereas ursolic acid reached its peak at Stage I.

### 2.2. Transcriptome Sequencing Analysis of Z. jujuba Mill. cv. Goutou Fruits at Different Ripening Stages

**Figure 2:**
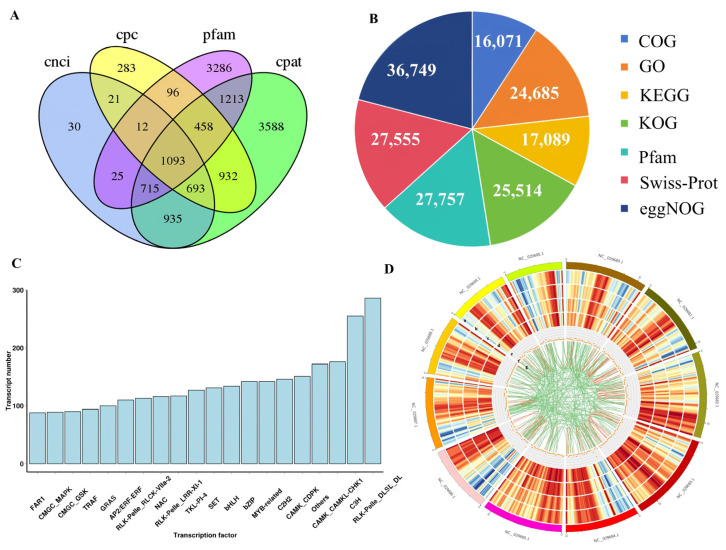
The transcriptome sequencing of *Z. jujuba* Mill. cv. Goutou fruits at seven ripening stages. (**A**) The Venn diagram of the four analysis results of lncrna. (**B**) The functional annotation of new transcripts. (**C**) The prediction of different types of transcription factors. (**D**) Circos diagram of differentially expressed genes: the data represented by the concentric circles from the outermost area to the innermost area are as follows: (a) chromosomes; (b) gene density within the genome; (c) gene density observed in the third-generation sequencing results; (d) transcript density within the genome; (e) transcript density observed in the third-generation sequencing results; (f) distribution density of LncRNA on chromosomes; and (g) distribution of fusion transcripts.

A total of 591,286 Read of Insert (ROI) sequences were obtained through the transcriptome sequencing of *Z. jujuba* Mill. cv. Goutou. After correcting the low-quality sequences with next-generation transcriptome data, the transcripts were aligned and subjected to redundancy analysis, resulting in 57,330 transcript sequences, which were assessed for completeness (Figure S2). Variable splicing analysis was performed on the deduplicated 57,330 transcripts, with 13,249 gene loci being identified, including 1827 novel gene loci and 38,450 newly discovered transcripts. The structural analysis of the newly identified transcripts predicted a total of 70,327 Signal Sequence Receptors (SSRs), 26,494 complete Open Reading Frames (ORFs), and 1093 Long Non-Coding RNAs (lncRNAs) (Figure 2A). Functional annotation was completed for 37,148 new transcripts (Figure 2B). We subsequently predicted the transcription factor in the transcript sequences, with the results including the AP2/ERF, bHLH, bZIP, NAC, and MYB families (Figure 2C). In addition, a Circos diagram was constructed to illustrate the density of the genes and transcripts, in addition to the distribution of different transcript types across chromosomes (Figure 2D).

**Figure 3:**
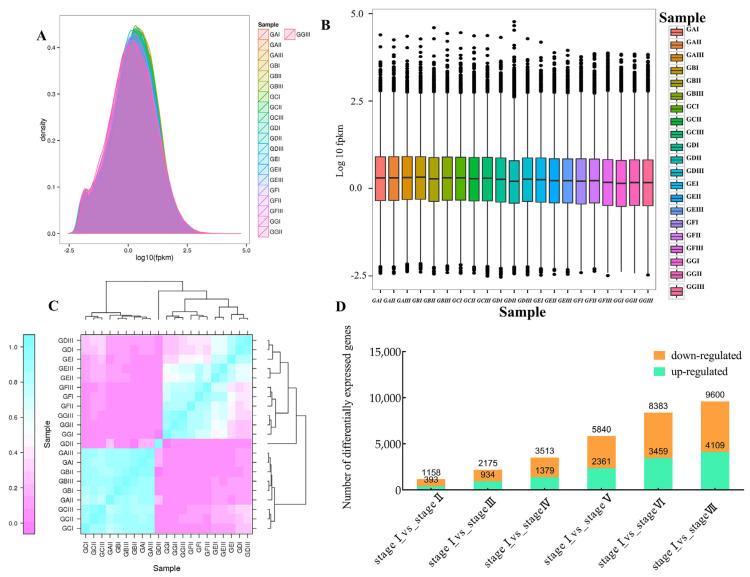
Analysis of differentially expressed genes in *Z. jujuba* Mill. cv. Goutou fruits at seven ripening stages. (**A**) The expression distribution of samples. GAI-GAIII: the samples at Stage I; GBI-GBIII: the samples at Stage II; GCI-GCIII: the samples at Stage III; GDI-GDIII: the samples at Stage IV; GEI-GEIII: the samples at Stage V; GFI-GFIII: the samples at Stage VI; GGI-GGIII: the samples at Stage VII. (**B**) The box plot of samples. (**C**) Thermogram of sample correlation: the Pearson correlation coefficient (r) was utilized as a metric for assessing biological replicate correlation. (**D**) Distribution of different genes at the different ripening stages.

We compared the transcriptome sequences with the reference genome and found that the number of reads mapped to the reference genome exceeded 80% in the clean reads, with the maximum percentage being 88.94% (Table S3). The overall distribution of transcripts was relatively uniform (Figure 3A), and the degree of scatter between samples was low (Figure 3B). From the correlation heatmap, we found that the correlation between the samples at Stage I to III was strong, and the samples at Stage IV to VII exhibited a good correlation (Figure 3C). Subsequently, a substantial number of differentially expressed genes were identified during the ripening of *Z. jujuba* Mill. cv. Goutou fruits through comparative analysis. Compared with Stage I, the number of differentially expressed genes, including upregulated and downregulated genes, exhibited an increasing trend at Stage II to Stage VII (Figure 3D).

### 2.3. Screening and Validation of Differentially Expressed Genes

Plant hormones play important roles in fruit ripening. For screening and validation, we performed functional annotations of the differentially expressed genes using GO and KEGG analyses. We identified 148 ethylene-related genes and 191 abscisic acid-related genes from the GO annotation of differentially expressed genes, of which were 43 candidate genes: CBL-interacting protein kinase 16 (*ZjCIPK16*), Cellulose synthase A catalytic subunit 8 (*ZjCesA8*), Galactinol synthase 2 (*ZjGOLS2*), NRT1/PTR FAMILY 5.2-like (*ZjNPF5.2-l*), delta 1-pyrroline-5-carboxylate synthetase (*ZjP5CS*), lipid phosphate phosphatase 2-like (*ZjEPHX2*), hypothetical protein L484_013434 (*ZjHP*), RING/U-box superfamily protein (*ZjPUB*), histidine kinase 2-like-1 (*ZjNME2-1*), NAC domain-containing protein 2 (*ZjNAC002*), fructose-1,6-bisphosphatase (*ZjFBPase*), Chitinase family protein (*ZjCHI*), Chitinase-like protein 1 (*ZjCHI1*), Enoyl-CoA hydratase/isomerase family (*ZjEci*), protein curvature thylakoid 1B (*ZjCURT1B*), 12-oxophytodienoate reductase 3-1 (*ZjOPR3-1*), 12-oxophytodienoate reductase 3-2 (*ZjOPR3-2*), diacylglycerol acyltransferase 1 (*ZjDGAT1*), uncharacterized protein (*ZjUP*), probable phospholipid hydroperoxide glutathione peroxidase (*ZjGPX*), plasma intrinsic protein 2 (*ZjPIP2*), CBL-interacting serine/threonine-protein kinase 1 (*ZjCIPK1*), ABC transporter G family member 36-1 (*ZjABCG36-1*), ATP-binding cassette transporter 2 (*ZjAbca2*), ABC transporter G family member 36-2 (*ZjABCG36-2*), Ammonium transporter 2 (*ZjamtB*), NAC domain-containing protein 72 (*ZjNAC072*), histidine kinase 2-like-2 (*ZjNME2-2*), NRT1/PTR FAMILY 5.2 (*ZjNPF5.2*), ACT domain-containing protein ACR8 (*ZjACR8*), auxin response factor 18-like (*ZjARF18*), glycine-rich RNA-binding protein GRP1A (*ZjGRP1A*), copper transporter 5.1 (*ZjCTR5.1*), LSD1-like protein (*ZjLSD1*), 24-methylenesterol C-methyltransferase 2 (*ZjSMT2*), multiprotein-bridging factor 1b-like (*ZjMBF1B*), GDSL esterase/lipase 2-like (*ZjGLIP2*), UBP1-associated protein 2C (*ZjUBA2C*), NAC domain-containing protein 8 (*ZjNAC008*), Serine/threonine-protein kinase-like protein ACR4 (*ZjACR4*), CBL-interacting serine/threonine-protein kinase 23 (*ZjCIPK23*), *ZjbHLH3*, and *ZjbHLH13* were selected for RT-qPCR. Through the KEGG pathway analysis, we found that eight hormones were associated with the seven ripening stages of *Z. jujuba* Mill. cv. Goutou fruits, with 127 differentially expressed genes related to plant hormone signaling pathways being selected. Among the differentially expressed genes, 40 genes are involved in the auxin signaling pathway, 18 genes are associated with the ABA signaling pathway, 9 genes are involved in the ethylene signaling pathway, 6 genes are involved in the GA signaling pathway, and 11 genes are involved in the JA signaling pathway. In this study, we selected 23 differentially expressed genes related to the ABA, ethylene, GA, and JA signaling pathways for validation using RT-qPCR. We compared the RT-qPCR and transcriptome data, excluding the *EIN3-1* and *ERF1/2* genes, and found that the expression levels of the remaining genes were consistent with the transcriptome data, thus verifying the accuracy of the transcriptome sequencing results.

### 2.4. Significant Differences in the Expression of Differentially Expressed Genes Involved in the ABA Signaling Pathway in Z. jujuba Mill. cv. Goutou Fruits at Different Ripening Stages

**Figure 4:**
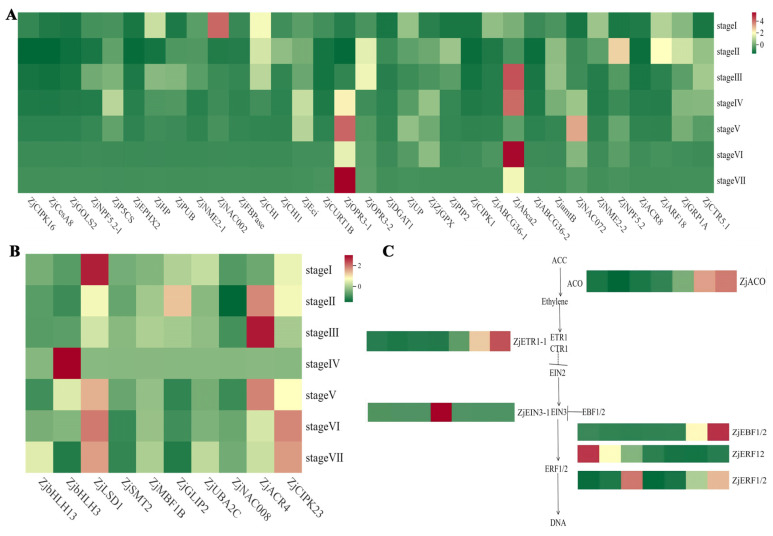
The expression of genes related to abscisic acid and ethylene in *Z. jujuba* Mill. cv. Goutou fruits at seven ripening stages. (**A**) The expression heatmap of genes related to abscisic acid in *Z. jujuba* Mill. cv. Goutou fruits at seven ripening stages. (**B**) The expression heatmap of genes related to ethylene in Chinese jujube fruits at seven ripening stages. (**C**) The expression levels of genes related to the ethylene synthesis pathway in *Z. jujuba* Mill. cv. Goutou fruits at seven ripening stages.

Based on the results of the transcriptome sequence of *Z. jujuba* Mill. cv. Goutou, we identified 33 genes related to the abscisic acid (ABA) synthesis and signaling pathway, with the results subsequently validated with RT-qPCR. The expression levels of *ZjABA1*, *ZjABA2*, *ZjABA6*, and *ZjABA9* decreased from Stage I to Stage VII, with the highest expression level at Stage I, and the expression levels of *ZjABA1*, *ZjABA2*, and *ZjABA6* significantly decreased at Stage II to Stage VII compared with those at Stage I. In contrast, the expression levels of *ZjABA3*, *ZjABA7*, *ZjABA11*, *ZjABA15*-*ZjABA17*, and *ZjABA33* increased significantly from Stage I to Stage VII, with the highest expression level at Stage VII. The expression levels of *ZjABA4*, *ZjABA10*, *ZjABA19*, *ZjABA20*, *ZjABA22*-*ZjABA25*, and *ZjABA27*-*ZjABA32* increased from Stage I to Stage VI, reaching a peak at Stage VI, and significantly decreased from Stage IV to Stage VII. The expression levels of *ZjABA5* and *ZjABA14* were highest at Stage V, with no significant differences between Stages V and VII. *ZjABA8* and *ZjABA26* showed the highest expression levels at Stage III. In addition, the expression levels of *ZjABA12*, *ZjABA13*, and *ZjABA21* peaked at Stage II. Lastly, *ZjABA18* exhibited the highest expression level at Stage VI (Figure 4A).

### 2.5. Expression Analysis of Differentially Expressed Genes Related to the Ethylene Signaling Pathway in Z. jujuba Mill. cv. Goutou Fruits at Different Ripening Stages

Based on the results of the transcriptome sequence of *Z. jujuba* Mill. cv. Goutou, we identified 10 genes related to the ethylene synthesis and signaling pathway, with the results subsequently validated with RT-qPCR. We found that the expression levels of *ZjETH1* and *ZjETH8* increased significantly from Stage I to Stage VI, followed by a decrease from Stage VI to Stage VII, peaking at Stage VI. In contrast, the expression levels of *ZjETH2*, *ZjETH4*, *ZjETH5*, *ZjETH6*, and *ZjbHLH13* significantly decreased from Stage II to Stage VII, with *ZjETH2*, *ZjETH5*, *ZjETH6*, and *ZjbHLH13* having the highest expression levels at Stage I, whereas *ZjETH4* had the highest expression level at Stage II, and the expression level of *ZjbHLH13* significantly increased from Stage V to Stage VII. The expression levels of *ZjETH3* and *ZjbHLH3* were highest at Stage IV, and *ZjETH7* showed the highest expression level at Stage III (Figure 4B). We selected six genes involved in the ethylene signaling pathway (Figure 4C), including *ZjACO*, *ZjETR1-1*, and *ZjEBF1/2*, which showed a significant increase in expression levels from Stage I to Stage VII, with the highest levels observed at Stage VII. *ZjEIN3-1* exhibited the highest expression level at Stage IV. In contrast, *ZjERF12* showed a significant decrease in expression level from Stage I to Stage VII, with the highest expression level observed at Stage I. *ZjERF1/2* exhibited the highest expression at Stage III.

### 2.6. Expression Analysis of DEGs Regulating the Jasmonic Acid and Gibberellic Acid Signaling Pathway in Z. jujuba Mill. cv. Goutou Fruits at Different Ripening Stages

**Figure 5:**
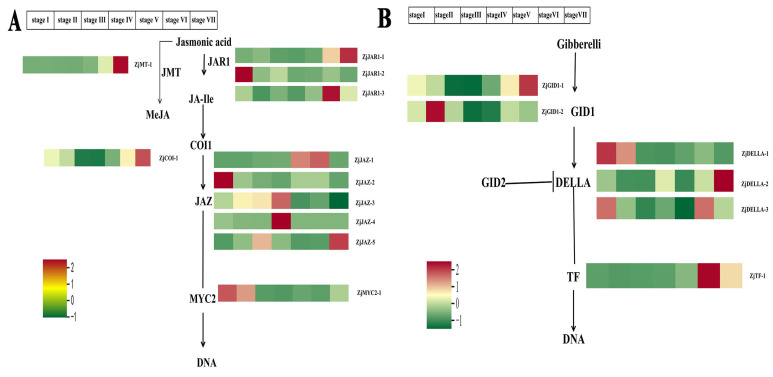
The expression levels of genes related to the jasmonic acid and gibberellin synthesis pathway in Chinese jujube fruits at seven ripening stages. (**A**) The expression levels of genes related to the jasmonic acid synthesis pathway in Chinese jujube fruits at seven ripening stages. (**B**) The expression levels of genes related to the gibberellin synthesis pathway in Chinese jujube fruits at seven ripening stages. JMT is the jasmonic acid carboxyl methyltransferase gene; TF stands for transcription factor.

Based on the results of the transcriptome sequence of *Z. jujuba* Mill. cv. Goutou, we identified 11 genes related to the JA synthesis and signaling pathway, with the results subsequently validated with RT-qPCR (Figure 5A). The results indicated that the expression levels of *ZjMYC2-1*, *ZjJAZ-2*, and *ZjJAR1-2* significantly decreased from Stage I to Stage VII, with the highest expression level being observed at Stage I. In contrast, compared with the other stages, the expression levels of *ZjJAR1-1*, *ZjCOI1-1*, *ZjJMT*, and *ZjJAZ-5* were significantly higher at Stage VII. The expression levels of *ZjJAR1-3* and *ZjJAZ-1* significantly increased from Stage I to Stage VI and peaked at Stage VI, followed by a significant decrease at Stage VII. Additionally, the expression levels of *ZjJAZ-3* and *ZjJAZ-4* showed a significant increase from Stage I to Stage IV, with the highest levels occurring at Stage IV and then decreasing from Stage V to Stage VII.

Based on the results of the transcriptome sequence of *Z. jujuba* Mill. cv. Goutou, we identified six genes related to the GA synthesis and signaling pathway, with the results subsequently validated with RT-qPCR (Figure 5B). The expression level of *ZjDELLA-1* decreased from Stage I to Stage VII, with the highest expression level observed at Stage I. *ZjDELLA-2* exhibited the highest expression level at Stage VII. The expression levels of *ZjGID-1*, *ZjTF-1*, and *ZjDELLA-3* reached their peak at Stage VI. However, *ZjDELLA-3* showed a decrease in expression level from Stage I to Stage V, followed by an increase at Stage VI before decreasing once again. *ZjGID1-2* exhibited the highest expression level at Stage II.

## 3. Discussion

After harvest and storage, fresh jujube fruits experience rapid water loss, shrinkage, softening, and rot, which negatively affects the quality and marketability of the fruit [25]. The developmental stage of jujube fruits is one of the factors that influences post-harvest storage and quality [26]. Plant hormones regulate the fruit development process through complex networks to slow fruit senescence, cracking, and softening, in addition to increasing the fruit set rate to improve fruit quality [9]. The results of previous studies have shown that the downregulation of ABA and ethylene biosynthesis prolongs the greenness of the fruit peel, with JA and ethylene contributing to fruit growth, and ethylene and ABA acting synergistically to accelerate fruit softening [27]. Therefore, the methods used in this study on plant hormones promoted fruit ripening and improved fruit quality.

As essential nutritional components for organisms, micronutrients play important roles in jujube growth. Cu is involved in the ethylene signaling pathway in the form of copper proteins, and its content fluctuations may be related to the regulation of ethylene metabolism [28]. In one study, during the fruit expansion phase, the application of exogenous Zn and naphthaleneacetic acid (NAA) increased the indole-3-acetic acid content of the fruit to accelerate cell division in the early stages of fruit ripening [29]. In this study, the Zn content of jujube fruits increased significantly from Stage I to Stage IV, suggesting its potential involvement in the fruit expansion process. Fe generally acts as a co-factor in the activity of redox enzymes that influence changes in fruit peel and pulp [30]. In this study, the Fe content of the jujube fruit showed significant differences between Stage IV and Stage V, with these differences being associated with changes in fruit peel color. Ursolic acid, betulinic acid, and oleanolic acid are three important and representative triterpenic acids found in jujube and accumulate during fruit ripening. ABA and JA are involved in the regulation of triterpenoid saponin synthesis and accumulation [31]. After binding to JAZ proteins and MYC2, JA regulates the expression of key genes involved in terpenoid biosynthesis that influence the promoters of downstream transcription factors and promote the synthesis and accumulation of oleanolic acid [32].

Plant hormones play important roles in fruit ripening. ABA is involved in plant gelation in response to abiotic stresses, fruit ripening, and processing leaf and fruit drop [33]. The ABA content is higher during the early stages of plant growth in order to promote plant growth. In this study, genes such as *ZjABA3*, *ZjABA4*, *ZjABA6*, *ZjABA7*, *ZjABA10*, *ZjABA11*, *ZjABA15-ZjABA19*, *ZjABA22-ZjABA25*, and *ZjABA27-ZjABA33* exhibited higher expression levels during the early ripening stages of jujube fruits, suggesting that ABA is involved in the regulation of the fruit growth process. In the late stage of fruit ripening, the ABA levels decrease in the phloem and ABA accumulates in the fruit, enhancing the fruit flavor, accelerating softening, and promoting color development [34]. In this study, the expression levels of the *ZjABA3*-*ZjABA7*, *ZjABA10*, *ZjABA11*, *ZjABA14-ZjABA17*, *ZjABA19*, *ZjABA20*, *ZjABA22-ZjABA25*, and *ZjABA27-ZjABA33* genes were high at Stage V, Stage VI, and Stage VII, indicating that they are involved in the drop of jujube fruits.

Ethylene regulates the expression of genes involved in fruit ripening and is involved in various biological processes [35]. The expression levels of *ZjETH1* and *ZjETH8* were positively correlated with fruit ripening, whereas the expression levels of *ZjETH2* and *ZjETH4* exhibited a negative correlation, suggesting that these genes are involved in fruit ripening. Additionally, the changes in *ZjbHLH3* and *ZjbHLH13* expression levels indicate that they participate in the color change of jujube fruits. Genes associated with the ethylene signaling pathway, such as *ZjACO*, *ZjETR1-1*, and *ZjEBF1/2*, showed a significant increase at Stage IV to Stage VII. The authors of previous studies have suggested that the ripening of jujube fruits may be regulated by *ZjACO*, which is involved in ethylene biosynthesis [36]. The trends in *ZjERF* genes aligned with changes in key genes involved in the ethylene signaling pathway [13]. Notably, *ZjERF12* exhibited a negative correlation with the expression levels of fruit ripening genes, suggesting that this gene plays a role in negatively regulating jujube fruit ripening. The results of previous studies have shown that the knocking down of *EIN2* inhibits the size and ripening of tomato fruits [37]. The expression level of *ZjEIN3-1* is associated with the accumulation of EIN3 in the ethylene signaling pathway, with EIN2 inhibiting the ubiquitination and degradation of the transcription factor EIN3. The high expression of *ZjEIN3-1* at Stage IV suggests that *ZjEIN3-1* may be involved in the ripening of jujube fruits.

JA coordinates with the ethylene signaling pathway and regulates fruit ripening by influencing ethylene [38]. The application of exogenous JA improved the metabolism of cell wall components to accelerate fruit ripening and softening [39]. Additionally, the coordination of MeJA and ethylene impacts the accumulation of yellow flavonoids and anthocyanins in pear peel to alter the color of pear peel [40]. *SmbHLH13* is involved in the JA signaling pathway and positively regulates the expression of *SmF3H* and *SmCHS*, which promote the anthocyanin biosynthesis of *Solanum melongena*. Additionally, in one study, *SmbHLH13* downregulated the expression of *SmFT*, which delayed flowering and influenced the changes in fruit color [41]. The expression level of *ZjJMT* significantly increased from Stage IV to Stage VII, indicating that it may participate in the ripening and color development of jujube fruit. In one study, ethylene interacted with JA to influence the expression of AP2/ERF transcription factors that were involved in fruit ripening, suggesting that JA influences fruit ripening through ethylene [42]. JA derivatives induced the expression of *ERF1* in the ethylene signaling pathway in one study to inhibit the biosynthesis of anthocyanins [43]. In another study, in the presence of ethylene, JA induced the biosynthesis of flavonoids/isoflavonoids, resulting in a deep yellow color in pear fruits [44]. The expression levels of *ZjJAR1-1*, *ZjJAZ-1*, and *ZjJMT* genes showed an increasing trend with jujube fruit ripening.

In one study, GA interacted with the key enzyme GID1 in the GA signaling pathway to bind to DELLA proteins and formed a GA-GID1-DELLA complex, which promoted the degradation of DELLA proteins via the ubiquitin–proteasome pathway and facilitated plant growth and development [45]. The results of previous studies have shown that DELLA proteins are involved in the regulation of various hormone signaling pathways, including ethylene, brassinosteroids, JA, and salicylic acid [46]. Following treatment with exogenous GA, tomato Nr mutants and *SlEBF3*-overexpressing lines delayed fruit ripening, indicating that the involvement of GA in fruit ripening is partially dependent on ethylene [47]. The antagonistic effect of rice *ACE1* and *DEC1* on GA regulated the growth of stems in rice in one study [48]. In this study, the expression levels of *ZjGID-1* and *ZjGID-2* showed a consistent increasing trend from Stage IV to Stage VI, suggesting that *ZjGID-1* and *ZjGID-2* may recognize and bind to GA in the fruit. This interaction probably alleviated the inhibitory effect on the downstream signaling pathways regulated by GA, with the downstream gene *ZjTF-1* also exhibiting an increasing trend at Stage IV to Stage VI. This finding is consistent with the role of the GA signaling pathway and suggests that GA may be involved in the regulation of jujube fruit ripening.

## 4. Materials and Methods

### 4.1. Plant Materials

Fruits of *Z. jujuba* Mill. cv. Goutou were collected in Yan’an, Shanxi Province, China (36°51′56″ N–110°21′13″ E), from July to September 2018. Jujube fruits were collected at seven ripening stages, based on previous studies [13], namely, Stage I (35 days after flowering (DAF)), Stage II (47 DAF), Stage III (61 DAF), Stage IV (78 DAF), Stage V (90 DAF), Stage VI (107 DAF), and Stage VII (119 DAF), from three 40-year-old trees. Each stage included three biological replicates. After removing the seeds, the fruits were dried and ground.

### 4.2. Transcriptome Sequencing and Functional Annotations

The jujube fruit samples at seven ripening stages (Stage I–VII) were sent to Beijing Biomarker Technologies (Beijing, China) for transcriptome sequencing. The SMARTer™ PCR cDNA Synthesis Kit (TaKaRa, code No. RR047A, Beijing, China) was utilized to synthesize full-length cDNA for transcriptome sequencing library construction. Third-generation sequencing (PacBio, Beijing, China) was combined with second-generation sequencing (Illumina, San Diego, CA, USA) to obtain a substantial number of raw reads from the cDNA library. HISAT2 was used to compare the clean reads and jujube genome [49], with positional information on the reference genome or genes being obtained. Expression levels of transcript genes were measured using FPKM (Fragments Per Kilobase of transcript per Million fragments mapped) with StringTie. Comparative analyses were performed to describe the overall distribution of transcript expression levels across the samples, with a Pearson correlation coefficient (r) r^2^ value closer to 1 indicating a stronger correlation between the two replicate samples. The degree of dispersion in individual sample expression levels was determined using density distribution plots and box plots and heatmaps of the correlation between samples. Once the expression levels of the genes had been predicted, assembled, and quantified, splicing diagrams were constructed from the read clusters, and individual transcript analyses were performed. During the differential expression gene detection process, DESeq2 was employed to perform differential expression analyses between the sample groups, yielding a set of differentially expressed genes between the two biological conditions. Fold change (FC) ≥ 2 and false discovery rate (FDR) < 0.01 were set as selection criteria. Differentially expressed genes were annotated using the KEGG Orthology (KO) and Gene Ontology (GO) databases.

### 4.3. Metal Element Analysis

Once the samples had been digested as previously described [2], an Inductively Coupled Plasma Mass Spectrometer (ICP-MS) (NexlON 2000, PerkinElmer, Shanghai, China) was used to determine trace metal element concentrations. The seven metal elements were quantified based on standard curves (Table S1).

### 4.4. Determination of Ursolic Acid, Oleanolic Acid, and Betulinic Acid Content

The 1 g *Z. jujuba* Mill. cv. Goutou samples were prepared with 25 mL of methanol. The mixture was kept in the dark overnight at 25 °C, and ultrasonication was performed for 40 min. The mixture was centrifuged for 10 min at 3000 rpm to collect the supernatant. After filtering with a 0.22 μm PTFE membrane, the samples were examined using high-performance liquid chromatography (HPLC). An Agilent Poroshell 120 EC-C18 column (4.6 × 150 mm, 4 μm) was used for separation. The mobile phase for oleanolic acid consisted of acetonitrile–0.2% phosphoric acid (90:10) at a flow rate of 1 mL/min, with detection at 205 nm. For ursolic acid and betulinic acid, the mobile phase consisted of acetonitrile–0.1% phosphoric acid (90:10, *v*/*v*) at a flow rate of 1 mL/min, with detection at 210 nm. The contents of Oleanolic acid, Betulinic acid and Ursolic acid in Chinese jujube fruit at different development stages (Figure S1). The column temperature was maintained at 27 °C, and the injection volume was 10 μL for all analyses. Ursolic acid, oleanolic acid, and betulinic acid concentrations were quantified based on standard curves (Table S2).

### 4.5. Real-Time Quantitative PCR (RT-qPCR) Analysis

Total RNA from the *Z. jujuba* Mill. cv. Goutou samples was extracted using the TaKaRa MiniBEST Universal RNA Extraction Kit (TaKaRa, Beijing, China). After evaluation of quality, 1 µg of RNA from each sample was subjected to first-strand cDNA synthesis using PrimeScript™ RT Reagent Kit with gDNA Eraser (TaKaRa, Beijing, China). Primer Premier 6.0 software was used to design the primers (Table S4). The total reaction system and RT-qPCR protocol was followed, as recommended for the TB Green Premix Ex Taq II kit (TaKaRa, Beijing, China).

### 4.6. Data Processing and Analysis

The data from the RT-qPCR were normalized using the 2^−ΔΔCT^ method with Stage I used as a reference, and the results of three biological replicates were analyzed. Statistical analysis of the fruits’ metal element, oleanolic acid, betulinic acid, and ursolic acid content and the RT-qPCR data was performed using one-way ANOVA with SPSS software (version 22.0) at the significance level of *p* ≤ 0.01. A correlation heatmap was generated to visualize the relationships between the samples, with only those with Pearson’s correlation coefficients > 0.8 and *p*-values < 0.05 being selected.

## 5. Conclusions

The ripening of jujube fruits is controlled by a complex network of multiple hormones. In this study, we analyzed the nutrient content and transcriptional expression levels of *Z. jujuba* Mill. cv. Goutou fruits at seven ripening stages. During Stage IV to Stage VII, there was an abundant accumulation of essential nutrients, including Zn, Fe, betulinic acid, and oleanolic acid. JA, ethylene, and GA were found to be primarily involved in the regulation of fruit ripening during this period, and the expression levels of the key genes, namely, *ZjERF1/2*, *ZjMYC2-1*, *ZjACO*, *ZjETR1-1*, *ZjEBF1/2*, *ZjERF1/2*, *ZjGID-1*, and *ZjTF-1*, significantly increased. ABA was found to mainly regulate the early stages of fruit ripening, with the expression of 22 *ZjABA* genes gradually decreasing from Stage I to Stage VII. The results of this study provide a theoretical basis for elucidating the regulatory mechanisms of jujube ripening and utilizing genetic engineering strategies to improve fruit quality. By investigating the relationship between hormone accumulation and fruit development, we can manipulate fruit flavor and color by regulating the expression of specific genes through hormone application. Furthermore, adjusting endogenous hormone levels can reduce the reliance on exogenous chemical hormones, promoting sustainable agricultural practices. The results of this study provide beneficial solutions for delaying fruit ripening, enhancing post-harvest preservation, and accelerating ripening, offering theoretical foundations and technical support for fruit tree breeding and fruit production. Furthermore, this study provides a research basis for improving nutritional value and fruit quality during the development of *Z. jujuba* Mill. cv. Goutou fruits.

## Data Availability

All relevant data are shown in the article and Supplementary Materials.

## Supplementary Materials

The following supporting information can be downloaded at https://www.mdpi.com/article/10.3390/ijms26083476/s1.
