# Supplementary material for: Transcriptome Screening of Hormone-Regulated Genes Related to Fruit Development in Zizyphus jujuba Mill. cv. Goutou Fruits at Different Ripening Stages
Source: Int J Mol Sci. 2025 Apr 8;26(8):3476. doi: 10.3390/ijms26083476 (PMC12026844; doi:10.3390/ijms26083476)
Supplement: Supplementary file 1 [file ijms-26-03476-s001.zip › ijms-3436381-supplementary.pdf]

Supplementary files

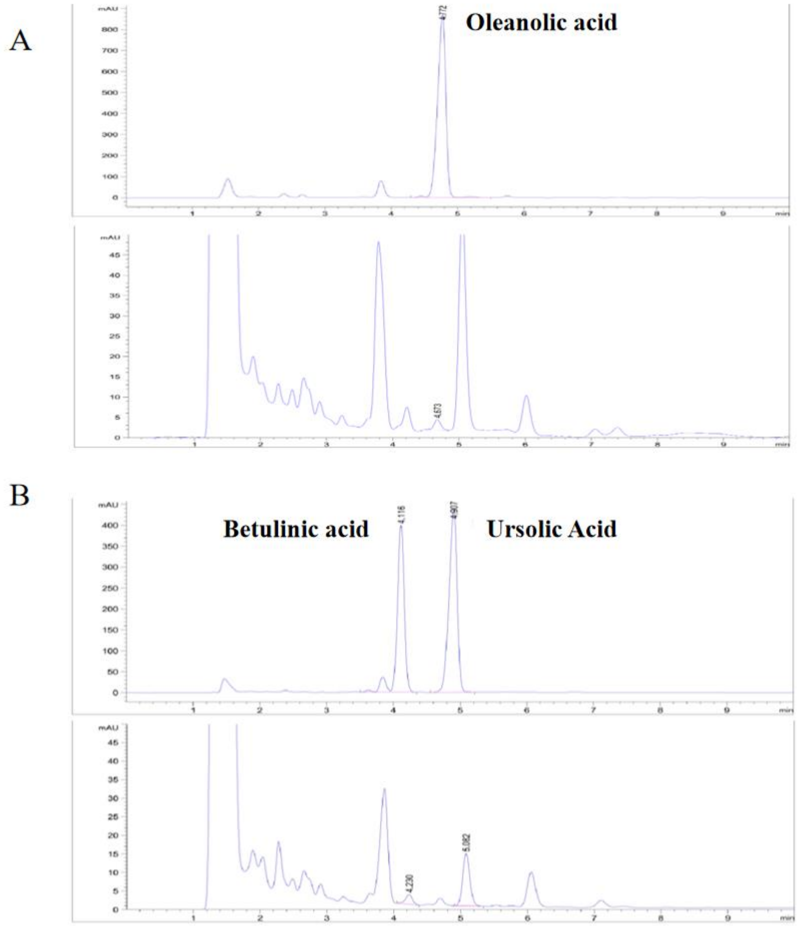

Figure S1. The contents of Oleanolic acid, Betulinic acid and Ursolic acid in Chinese jujube fruits at different ripening stages.

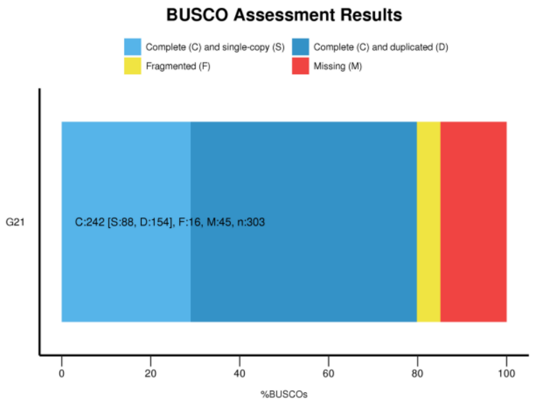

Figure S2. BUSCO integrity assessment.

Table S1. The standard curve of seven elements of Chinese jujube fruits at different ripening stages.

Element	Mass number	Equation	Correlation coefficient	BEC（μg/L）
Mg	24	y=165x+0	0.999855	9.777991
Al	27	y=39x+0	0.999893	7.695274
Ca	40	y=4x+0	0.999963	5.711899
Mn	55	y=660x+0	0.999963	0.134755
Fe	56	y=41x+0	0.999961	3.425946
Cu	63.5	y=3822x+0	0.999864	0.237816
Zn	65	y=245x+0	0.999949	2.111478

Table S2. The standard curve of three Triterpenic acid of Chinese jujube fruits at different ripening stages.

Compound	Equation	Correlation coefficient
Ursolic Acid	y = 4758.5x + 28.706	0.9994
Betulinic acid	y = 3055.9x - 8.5135	0.9993
Oleanolic acid	y = 7485.1x + 29.571	0.9998

Table S3. The results of RNA-Seq data and reference to genome.

Sample	Clean Reads	Mapped reads	Uniquely mapped reads	Multiple mapped reads
GAI	52963406	87.34%	75.91%	11.43%
GAII	47599514	86.82%	75.66%	11.16%
GAIII	49950122	84.09%	73.28%	10.81%
GBI	50300712	86.01%	74.33%	11.69%
GBII	52868830	87.56%	75.59%	11.97%
GBIII	51965042	86.53%	74.62%	11.92%
GCI	45509352	87.07%	74.99%	12.08%
GCII	54084266	87.29%	75.32%	11.97%
GCIII	62540806	87.53%	75.39%	12.14%
GDI	72258026	87.33%	76.18%	11.16%
GDII	53800798	86.62%	74.92%	11.70%
GDIII	55622466	87.91%	76.74%	11.17%
GEI	63026510	87.73%	76.95%	10.78%
GEII	58905236	88.08%	77.20%	10.89%
GEIII	59868308	87.18%	76.37%	10.81%
GFI	53128872	88.94%	77.90%	11.04%
GFII	59365386	87.10%	76.16%	10.93%
GFIII	66125710	87.76%	75.92%	11.84%
GGI	63500626	81.53%	71.01%	10.52%
GGII	58847296	84.07%	73.38%	10.69%
GGIII	60450036	86.08%	75.07%	11.01%

Note：GAI- GAIII: The samples of stage I, GBI- GBIII: The samples of Stage II; GCI- GCIII: The samples of Stage III; GDI- GDIII: The samples of Stage IV; GEI- GEIII: The samples of Stage V; GFI- GFIII: The samples of Stage VI; GGI- GGIII: The samples of Stage VII.

Table S4. Primer sequences.

Genes	Primer5'to 3'
ZjActin-F	AGCCTTCCTGCCAACGAGT
ZjActin-R	TTGCTTCTCACCCTTGATGC
*ZjbHLH13*_F	CCGTCCGATGAGTGCTGAATCC
*ZjbHLH13*_R	TCTCTTCCATTGGCAGGCTTCC
*ZjbHLH3*_F	TGGTCCTGGCGAGGCATACAA
*ZjbHLH3*_R	ACAGGCACCAGCACTACAGTCT
*ZjJAZ-4*_F	GGCGGCCAAGTGTTGGTGTT
*ZjJAZ-4*_R	GGCTTGAGGTCGTCGTTGAAGG
*ZjCOI1-1*_F	CCAGAGCCGCCATGTTCAATCT
*ZjCOI1-1*_R	AACGAGCCAGGAGTTCAAGGTC
*ZjJAZ-5*_F	CCAGAAGCGACAGCCAAGCA
*ZjJAZ-5*_R	GCAGCCTGCGGCACAAACTA
*ZjJAZ-3*_F	GGCTCAAGTGCAGGCACCATT
*ZjJAZ-3*_R	TGGAACCACGGTTGTTGTAGCA
*ZjJAZ-1*_F	CGGGAGCGGTACAAACGACAGA
*ZjJAZ-1*_R	TCCTTCTTCCCTCGGCGTCTCT
*ZjJAR1-3*_F	GGTATTGTCCTTGCTCGCTGCT
*ZjJAR1-3*_R	TCTTGGCTCTGCTTGGCTAACG
*ZjJAR1-2*_F	AGGTGACAGGCTTCCACAACTC
*ZjJAR1-2*_R	CCTCACTTCCTTCGCCACTCAA
*ZjJAR1-1*_F	TGTTGCCGACGAATGAATGTGT
*ZjJAR1-1*_R	TGAGTTGTGGAAGCCTGTTACC
*ZjMYC2-1*_F	TACCACCACCACCACCACCATC
*ZjMYC2-1*_R	AGCGTTGTCGTCAGTCCAGAGA
*ZjJAZ-2*_F	CAGAGCCACGCCACTTATCCTT
*ZjJAZ-2*_R	AGTTTGGTTGGGAGGCAGAGTC
*ZjGID1-2*_F	TGAGAAGCCTGTGACCTCTGAG
*ZjGID1-2*_R	CGATAGTTGACCGAGACGACCA
*ZjDELLA-3*_F	AAGCCTTGGCGGTCAACTCC
*ZjDELLA-3*_R	GCTCGCTTCCTGCTCTACAACA
*ZjGID1-1*_F	CGTGGTCTGTCTGAGCGGTTCT

*ZjGID1-1*_R	CGATGTGGGAAGGCGACGACTA
*ZjTF-1*_F	GCTGCTGCTGCTGCTACCAA
*ZjTF-1*_R	AGAGTGCTCATCGGCTGGAACA
*ZjDELLA-1*_F	ACATAGGCTTGCTCGCTTCGT
*ZjDELLA-1*_R	AGAATGGCTTGGTTGGCTGTGA
*ZjDELLA-2*_F	GCTTCCGACACCGTTCACTACA
*ZjDELLA-2*_R	TCGCCACGCTGGATGGATTC
*ZjEBF1/2*_F	GGGATGGGTGGGTTTGGTTTGA
*ZjEBF1/2*_R	AGGCACAGGCTTCTTGAACAGA
*ZjERF1/2*_F	TGACCAAGCCGCATTCGCAAT
*ZjERF1/2*_R	CCAAGTCCTCCAACACCACCAC
*ZjEIN3-1*_F	ACGGTCAAGGAGTTGGATTGGA
*ZjEIN3-1*_R	TGCTGAGGTTGTGGTTGTGGTT
*ZjETR1-1*_F	GCAACTGTGACGACGAAGGTAG
*ZjETR1-1*_R	ACGGTGAGAGCCAGCATTAGC
*ZjSAMT*_F	ATCGGCTGGAAGTTTCGGAGGT
*ZjSAMT*_R	ATGCACTTGGCGACGCTGTATC
*ZjJMT*_F	AGCCAGTGATTGAGGAAGCCAT
*ZjJMT*_R	GAGCAGCCTAAGGAGCAGCATT
*ZjCIPK16*_F	CCCAGTCCAAGCACCAGAATCG
*ZjCIPK16*_R	TGCCACGGTAGTCGGATGTTGA
*ZjCesA8*_F	GCGTTGGAGCGGTGTTGGTATC
*ZjCesA8*_R	TGGCGGTGACAGTGAAGTTGGT
*ZjGOLS2*_F	CCACATACCACGACCTCCTCAA
*ZjGOLS2*_R	GCAGCACAGTAATGGACCACCT
*ZjNPF5.2-l*_F	AGAGGCAATGTACCGTGTGGAA
*ZjNPF5.2-l*_R	TGGCTGGGTTGACAAGTGGATG
*ZjP5CS*_F	GGTCCAAGAAGCGGCAGTATCA
*ZjP5CS*_R	AAGACGGCGACTCAGTAGGC
*ZjEPHX2*_F	GCGTTGTTCATCTTGCGTCAGA
*ZjEPHX2*_R	CGAGGACGACCAACAGCATCT
*ZjHP*_F	TGAGTGGCTGCAAAGTGGGAAT
*ZjHP*_R	GCTGCTCGTGCTGCTACTATTG

*ZjPUB*_F	ACGGCACTGTCCATCTCCATAG
*ZjPUB*_R	TCGTATGATTCGGAAGGGTGGT
*ZjNME2-1*_F	GCGAGCAAGGGCAACTGGAA
*ZjNME2-1*_R	GCGGTAGCATCTGAAGGAAGGT
*ZjCTR5.1*_F	CTACTGGAGCAAGCAGGTGACC
*ZjCTR5.1*_R	GGAAGCAAGTTTGAGGCGGAGT
*ZjNAC002*_F	ACACAAGGACGGAACGCCATT
*ZjNAC002*_R	CGTGTGAAGCCTCGGGATAGAT
*ZjFBPase*_F	GTCGTCCGAAACTTCCCTCCAA
*ZjFBPase*_R	GGTGGTGGTGGTGGTAATCAGT
*ZjCHI*_F	CGACGAGGTTGGTGAAGAAGGT
*ZjCHI*_R	CGGAGAGTTGCGCTTGGAGAA
*ZjCHI1*_F	AGAGATGACTTGCGGACAGG
*ZjCHI1*_R	TCAGCACAGGATAGCACTTCA
*ZjEci*_F	TGCCAACCATCGCCGCCTTA
*ZjEci*_R	ACCTCATCGCCACCGTGAAGTA
*ZjCURT1B*_F	CTCCACCTCCAGTCCAGTCTCA
*ZjCURT1B*_R	CGGGCAACCTTACGACCTGAAA
*ZjOPR3-1*_F	TCAGCGAAGGCACTTCTCCATC
*ZjOPR3-1*_R	GGCGTGAACAGCATCTACAACC
*ZjOPR3-2*_F	CGTGTTCATCGTGTCGTGGGTA
*ZjOPR3-2*_R	AGTGGCTGTGTTGGTCGGTATG
*ZjDGAT1*_F	GCGATTGCGATTGCGAGGATG
*ZjDGAT1*_R	TCTTATGAGCAGGAGCCGATGG
*ZjUP*_F	CGGCTAACTTAGGCGGCGTAAC
*ZjUP*_R	TCCTCAAGCCACTGCCATCCAA
*ZjGPX*_F	CCCACCACCAGCTTTCACAAAT
*ZjGPX*_R	CCAAGCAGGTCAACAGGAACAC
*ZjPIP2*_F	CCGCTAATCGACGCCGAAGAAT
*ZjPIP2*_R	CCAACACCGCCGCAATCATCT
*ZjCIPK1*_F	GCGTAGCCTGCCTTGTTGAATC
*ZjCIPK1*_R	TCCGAATGGGTTTCCACTTTGC
*ZjABCG36-1*_F	GCAGAAGCGTAAGCAGAAGCAT

*ZjABCG36-1*_R	CACTGGTCCGAAGCCGATCAT
*ZjAbca2*_F	GGCACAATCTTCTGGCAGGTT
*ZjAbca2*_R	TCCAACGAACACTACCGCTACA
*ZjABCG36-2*_F	GCAGAAGCGTAAGCAGAAGCAT
*ZjABCG36-2*_R	CACTGGTCCGAAGCCGATCAT
*ZjamtB*_F	GGTGGTGGCTGCTTTGTTTGTG
*ZjamtB*_R	CTCCATGAACGGCGTCATCTCC
*ZjNAC072*_F	CGTGGACTCGCCGTTGAAGA
*ZjNAC072*_R	CTGTTGGAGTTGGGCTGGAAGA
*ZjNME2-2*_F	GCGAGCAAGGGCAACTGGAA
*ZjNME2-2*_R	GCGGTAGCATCTGAAGGAAGGT
*ZjNPF5.2*_F	CACCGTCAGCTCTGCCAACAA
*ZjNPF5.2*_R	GTCCAGTAGCGACCGAGATGAG
*ZjACR8*_F	GCTGGCAGGCAGAGAAGAAGA
*ZjACR8*_R	ACCTCACCGAGCAGACAATGAA
*ZjARF18*_F	CGGTGAGAGCGGCAATGAGGAT
*ZjARF18*_R	CCAGCGGATTGGATCAGCAACC
ZjGRP1A _F	GCCGCAATATCACCGTGAACGA
ZjGRP1A _R	AACCACCACCACCTCCATAGCC
ZjLSD1-F	TCCGTGTTTGTCTCGGAGGCAT
ZjLSD1-R	TTGGTGGTAGAGAAGCCGCAGG
ZjSMT2-F	CAGCCGTGGTGGACAAGGTTGA
ZjSMT2-R	AAGGCCGCGAGCACAGTGACTA
ZjERF12-F	AGCCGCCTCCGTTATGGATGTG
ZjERF12-R	AGGTGAGAGACGCCGCCGTAAA
ZjMBF1B-F	TGGCCGGAGTTGGACCGATTTC
ZjMBF1B-R	TCGTGCGGCGTTGACAGCTT
ZjGLIP2-F	TGCTGTGGCAGTGGACCCTTCA
ZjGLIP2-R	AGGCCCTTTCAGTGGGATGAGC
ZjUBA2C-F	CCCACCGCCACCACAAACACAA
ZjUBA2C-R	TGGAGGAGCTTGCGTGCGTCTT
ZjNAC008-F	TGCTGCTGTGTGTTGCTGCGTA
ZjENAC008-R	ACGTCACTGTCCCATGCCATGC

ZjACR4-F	TCCGGTCAAGGCTTGTGCTCAT
ZjACR4-R	ACGGCGGCGTAGACTGTTCCAA
ZjCIPK23-F	GCGGACGAGCAGCAGCAACAAT
ZjCIPK23-R	TCCGAGCGAACTTGACCTTGGC
ZjACO-F	TTCGAGCCCACACCGATGCA
ZjACO-R	CGATGGAGTGACGCATTGGTGG
